# Glycemic control in children with type 1 diabetes treated with the advanced hybrid closed loop system 2-year prospective, observational, two-center study

**DOI:** 10.3389/fendo.2024.1332418

**Published:** 2024-02-08

**Authors:** Sebastian Seget, Agata Chobot, Mateusz Tarasiewicz, Anna Bielawska, Ewa Rusak, Agnieszka Ochab, Joanna Polanska, Przemysława Jarosz-Chobot

**Affiliations:** ^1^ Department of Children’s Diabetology, Medical University of Silesia, Katowice, Poland; ^2^ Department of Pediatrics, Institute of Medical Sciences, University of Opole, Opole, Poland; ^3^ Department of Data Science and Engineering, Silesian University of Technology, Gliwice, Poland

**Keywords:** advanced hybrid closed-loop system, BMI, body mass index, children, time in range, type 1 diabetes

## Abstract

**Background and aims:**

MiniMed 780G is the first Advanced Hybrid Closed Loop (AHCL) system in Poland, approved in the EU in 2020. To date, observations of glycemic control up to 12 months have been published. This study aimed to analyze glycemic control and anthropometric parameters in children and adolescents with type 1 diabetes (T1D) after two years of using the AHCL system.

**Materials and methods:**

We prospectively collected anthropometric data, pump, and continuous glucose records of fifty T1D children (9.9 ± 2.4 years, 24 (48%) boys, T1D for 3.9 ± 2.56 years) using an AHCL system. We compared the two-week AHCL records obtained after AHCL enrollment with data 6, 12, and 24 months after starting AHCL.

**Results:**

Time in range (70-180 mg/dl) and BMI z-score did not change during the 2 years of observation (p>0.05). The percentage of autocorrection in total daily insulin increased significantly (p<0.005).

**Conclusion:**

Glycemic control in the investigated group of children with T1D treated with the AHCL system for 2 years remained stable. Children in this group maintained weight and optimal metabolic control, most likely due to autocorrection boluses.

## Introduction

Type 1 diabetes (T1D) is children’s and adolescents’ most common glucose metabolism disorder. Through the years, enormous progress has occurred in treatment and monitoring options. Multiple daily injections (MDI) evolved into continuous subcutaneous insulin infusion (CSII), and continuous glucose monitoring (CGM) systems brought a revolution in glucose monitoring. Technology helped to reduce the severity and then the frequency of hypoglycemia episodes by the use of low glucose suspend (LGS) and predictive low glucose suspend (PLGS) devices (1). Another crucial moment was implementing automated insulin delivery (AID) systems, including the advanced hybrid closed-loop (AHCL) system. The first AHCL system in Poland was Minimed 780G Medtronic approved in the European Union in June 2020 (2). The AHCL system automatically adjusts basal insulin infusion to the current glucose level together with autocorrection dosing (3).

Studies present that using the AHCL system remarkably improves glycemic control in patients with T1D, primarily by increasing the time spent in tight glucose range of 70-140 mg/dl (TITR). It stabilizes overnight glucose levels without significantly increasing hypoglycemia episodes (3). The use of AHCL systems provides more stable glycemic control, and we can expect that it improves the quality of life of children with T1D and their families. Some research concludes that in patients where an AHCL system replaced the LGS or PLGS system, glycemic control significantly improved simultaneously with increased everyday life quality and psychosocial well-being (4, 5). AHCL systems assume a reduction in the frequency of interference in insulin therapy from the site of the person living with diabetes and the family. The aim of these systems is to make treatment more comfortable with unchanged or even improved glycemic control. Insulin delivery using MiniMed 780G gives a chance to simplify insulin delivery for meals and physical activity (6–8).

We present a 2-year prospective, observational, two-center research study focused on anthropometric data and glycemic control parameters of children and adolescents with T1D using an AHCL system. For today, this is the most extended observation presented in a publication. This study continues the previous 12-month observation of the same cohort of children with T1D (9).

## Methods

We followed prospectively 50 children and adolescents with T1D using the MiniMed 780G system, who are under the care of two regional pediatric diabetes centers (Department of Children’s Diabetology, University Clinical Hospital of the Medical University of Silesia in Katowice and Department of Pediatrics, University Clinical Hospital of the University of Opole, Poland), both Centers of Reference of the SWEET (Better control in Pediatric and Adolescent diabeteS: Working to crEate CEnTers of Reference) network. All studied patients were changing from a pump with predictive low glucose suspend (PLGS) to an AHCL system. The inclusion criteria for the study were age ≦18 years, more than 70% of the sensor wear and usage time, and more than 70% spent in the automatic mode. The mentioned criteria were necessary to correctly interpret continuous glucose monitoring (CGM) and AHCL data. In most children and adolescents with T1D treated with the MiniMed 780G system in automatic mode, we set the most satisfactory settings - active insulin time 2 hours, target 100 mg/dl. The study group was characterized by biometric parameters - age, sex, and duration of T1D. Data from the AHCL system was automatically sent to the CareLink server and retrieved using CareLink Professional software (Medtronic MiniMed, USA). Two-week AHCL records and anthropometric parameters - body mass and height - were collected prospectively right after AHCL enrollment, then 6, 12, and 24 months after starting AHCL. For each time point, we calculated the body mass index (BMI) z-score using the individual’s weight and height and the World Health Organization (WHO) reference values (10). CGM readings were analyzed using GlyCulator 3.0 software (Medical University of Łódź, Poland) (11).

The analysed parameters were summarised by their mean value and standard deviation. The box plots and time plots for each variable were constructed. The horizontal line inside the box on the boxplot represents the median value; upper and lower box edges relate to the upper and lower quartiles, while whiskers reach the minimum and maximum values in the data. Each patient-related time series is represented by a set of points connected by a dotted line and colour-coded.

Shapiro-Wilk test was used to check distribution normality. Friedman’s repeated measures ANOVA was applied to verify the hypothesis on mean value stability in time. The ANOVA-based time series analysis was supported by the Jonckheere-Terpstra test for trend and Kendall’s concordance coefficient W as a measure of effect size. W value less than 0.2 was interpreted as slight agreement in time response, in the range of [0.2, 0.4) as fair agreement in time response, [0.4, 0.6) - moderate agreement in time response, [0.6, 0.8) - substantial agreement in time response, and 0.8 and more as almost perfect agreement in time response. No correction for multiple testing was applied. P-values less than 0.05 were treated as significant.

## Results

The study included 50 pediatric participants with T1D (24 (48%) boys), with an average age of 9.9 ± 2.4 years and an average duration of the disease of 3.9 ± 2.56 years (parameters are reported at the beginning of the 24-month observation). **Table 1**. presents data from the AHCL, including insulin infusion and sensor data.

**Table 1:** Daily insulin doses and sensor data of children and adolescents with type 1 diabetes using the advanced hybrid closed-loop system at the initiation of the system and after 6, 12, and 24 months of follow-up.

	The first 2 weeks of AHCL start	After 6 months	After 12 months	After 24 months	Friedman ANOVA p-value (P_A_), Jonckheere-Terpstra test p-value (P_T_), and Kendall concordance coefficient (W)
Avg SG [mg/dl]	131.36 ± 11.04	132.46 ± 11.73	132.45 ± 13.42	136.09 ± 13.62	P_A_=0.041453 P_T_=0.020450 W=0.05
GMI [%]	6.45 ± 0.26	6.48 ± 0.28	6.48 ± 0.32	6.57 ± 0.33	P_A_=0.041453 P_T_=0.020396 W=0.05
CV [%]	34.99 ± 5.17	33.75 ± 5.02	34.06 ± 5.38	33.93 ± 9.86	P_A_=0.646760 P_T_=0.152809 W=0.01
TDI [u/kg]	0.67 ± 0.20	0.77 ± 0.22	0.79 ± 0.20	0.84 ± 0.22	P_A_<0.000001 P_T_=0.000157 W=0.34
Basal insulin [%]	34.88 ± 6.91	33.16 ± 5.09	35.08 ± 6.36	34.82 ± 6.21	P_A_=0.012351 P_T_=0.496950 W=0.07
Bolus insulin [%]	65.06 ± 6.90	66.84 ± 5.09	64.92 ± 6.32	65.17 ± 6.21	P_A_=0.008158 P_T_=0.463885 W=0.08
Autocorrection [%]	11.24 ± 6.08	11.94 ± 5.63	12.40 ± 5.79	14.61 ± 7.00	P_A_=0.002034 P_T_=0.001934 W=0.10
Using the sensor [%]	95.10 ± 3.94	94.04 ± 4.69	93.80 ± 4.53	95.66 ± 3.66	P_A_=0.002097 P_T_=0.088520 W=0.10
Auto mode [%]	94.26 ± 6.28	96.62 ± 5.70	97.56 ± 2.79	97.58 ± 3.21	P_A_=0.005793 P_T_=0.02979 W=0.08
Percentage of sensor glucose values in range					
>250 mg/dl [%]	2.33 ± 2.52	2.34 ± 2.31	2.68 ± 3.48	3.10 ± 3.11	P_A_=0.566952 P_T_=0.080732 W=0.01
180 - 250 mg/dl [%]	13.13 ± 5.74	12.83 ± 5.88	12.59 ± 5.78	14.21 ± 6.16	P_A_=0.061049 P_T_=0.162546 W=0.05
70 - 180 mg/dl [%]	79.28 ± 8.12	81.16 ± 7.83	80.40 ± 8.25	79.33 ± 8.19	P_A_=0.362959 P_T_=0.432356 W=0.02
54 - 70 mg/dl [%]	4.15 ± 2.70	2.95 ± 1.76	3.40 ± 2.34	2.79 ± 2.09	P_A_=0.105073 P_T_=0.010752 W=0.04
<54 mg/dl [%]	1.11 ± 1.07	0.73 ± 0.77	0.93 ± 0.92	0.58 ± 0.70	P_A_=0.174635 P_T_=0.028549 W=0.03

Avg SG, average sensor glucose; CV, coefficient of variation; GMI, glucose management indicator; TDI, total daily insulin. P-values < 0.05 are treated as significant. The measurements are represented as the mean value ± standard deviation.

**Figure 1:**
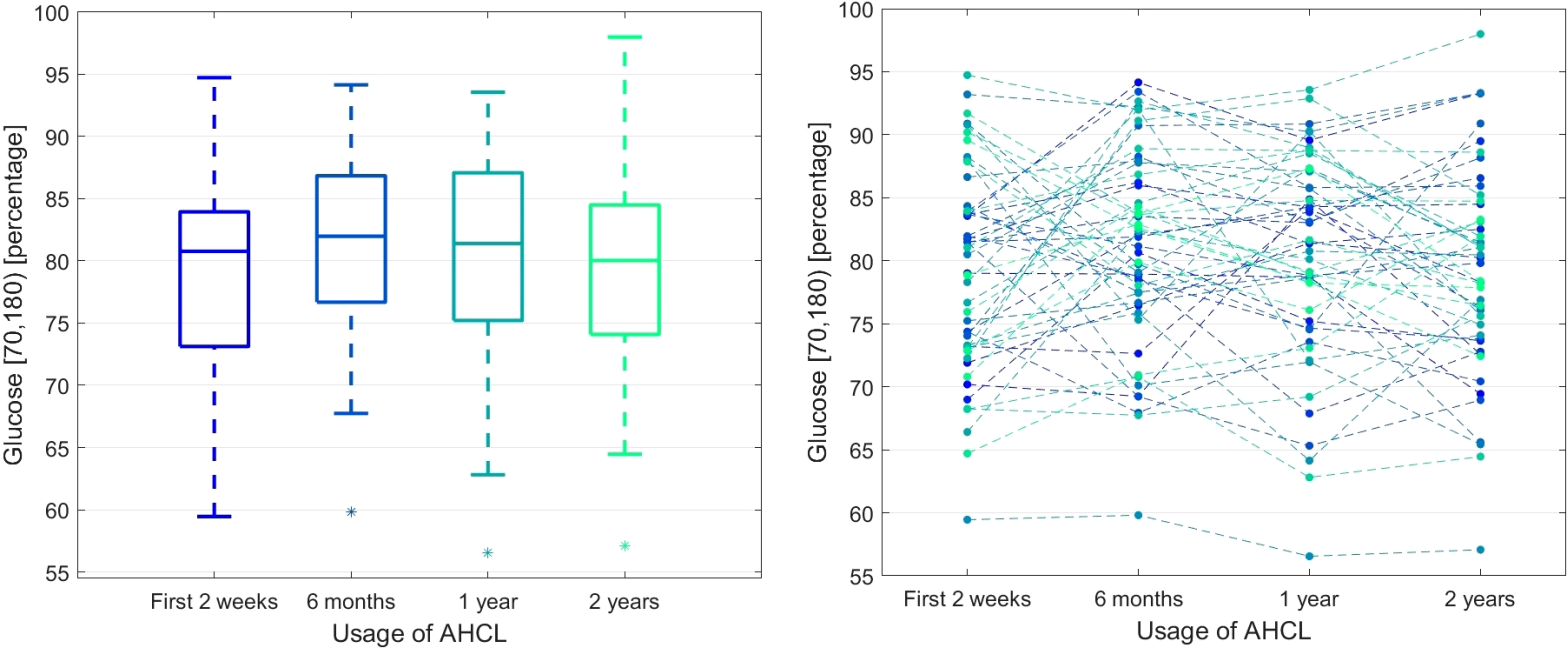
The percentage of glucose measurements in the 70-180 mg/dl range during the observation time. The left panel presents the box plots independently calculated for each observation time point, while the right panel shows the time series for each patient.

Time series analysis by Friedmann ANOVA showed significant changes in the GMI and average SG in time (p_A_ < 0.05). The *post hoc* comparisons revealed a slight increase in GMI and average SG after two years, but the overall response pattern was not very similar across all patients (W < 0.2). Still, the time spent in specific glycemic ranges did not change significantly between individual observation periods (p_A_ ≥ 0.05). **Figure 1** demonstrates the data for the glycemic range of 70-180 mg/dl.

**Figure 2:**
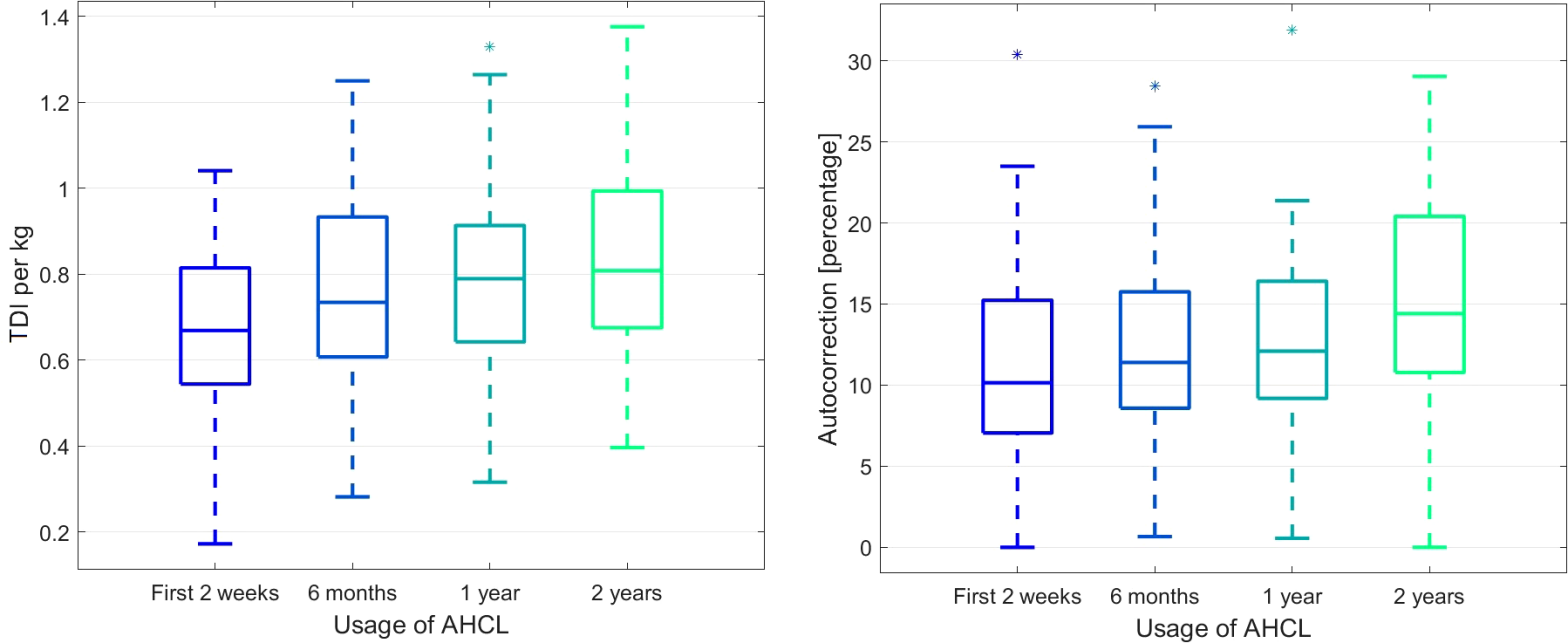
The boxplots for the Total Dose of Insulin (TDI) per kg (left panel) and the proportion of the autocorrection in TDI during the time of observation (right panel).

After two years of using the AHCL system, the use of the sensor and auto mode slightly increased (p_A_ < 0.05). We observed that after using the Minimed 780G system in auto mode, TDI per kg significantly and consistently increased (p_A_ < 0.000001 and W = 0.34, fair agreement) with synchronous increase of the proportion of autocorrection in TDI (p = 0.002034, W = 0.10, slight agreement across patients). **Figure 2** visualises the data.

**Figure 3:**
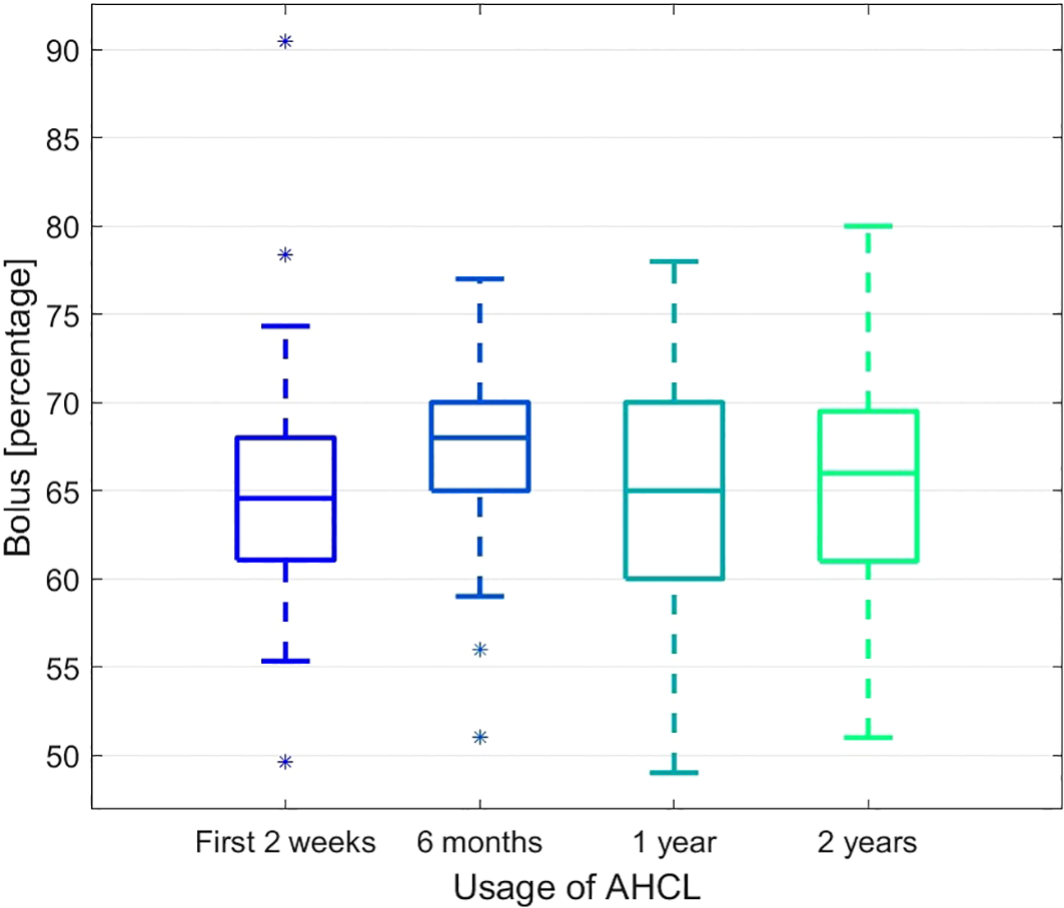
The boxplot presenting the changes in time for the percentage of the bolus insulin.

The changes in time in the percentage of bolus and basal insulin were also observed. The proportion of the bolus insulin increased significantly after 6 months but then stabilised and remained unchanged after two years when compared to the starting time point (see **Figure 3**). The complementary process is observed for the basal insulin.

**Figure 4:**
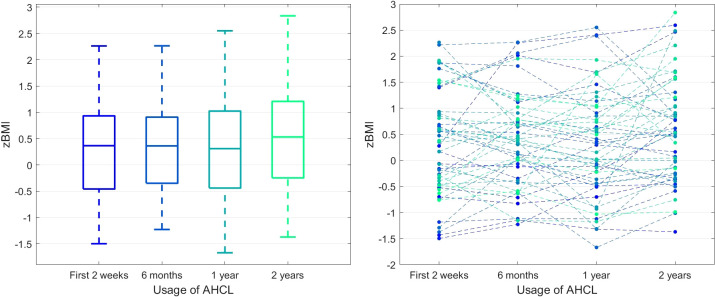
BMI z-score in time. The left panel presents the box plots independently calculated for each observation time point, while the right panel shows the time series for each patient.

Finally, we observed that the BMI z-score values were stable at subsequent time points (p_A_ = 0.1305). **Figure 4** represents the details of that process. At the same time, the average value of carbohydrates from one day entered into the system in individual 2-week periods systematically increased (184.74, 206.44, 203.10, 215.20, p_A_ = 0.0021, test for trend p_T_ = 0.0288).

## Discussion

The results revealed several interesting observations. Firstly, a significant increase in autocorrection and total daily insulin doses accompanied the increasing use of the auto-mode. We also observed that the use of the sensor remains high and unchanged, which is necessary for the proper work of the SmartGuard mode and to allow the system for a more patient-independent insulin delivery. As shown by Tornese G. et al., autocorrections allow a more liberal approach to dietary aspects of the therapy. The authors revealed that in case of not very exact carbohydrate counts or a missed meal bolus, the AHCL system helped by delivering an autocorrection dose without the patient’s intervention (6). Similar outcomes presented a study comparing glycemic control for announced and unannounced meal challenges. Unannounced meals containing up to ≤80 g of carbohydrates caused the glycemia level to rise no higher than 250 mg/dl. There was no difference in TIR in the announced and unannounced ingestion of less than 20 g of carbohydrates (12). Rachmiel M. et al. proved that using the AHCL system increases the TIR in youths with T1D despite less compliance (13). Autocorrection would likely improve glycemic control, particularly in the case of patients who miss more or give delayed or inaccurate insulin boluses. Therefore, the AHCL system may be beneficial not only for people carefully following diabetes self-care but also for those who less accurately assess food or forget to bolus (for meals and corrections). It has to be mentioned that the use of the AHCL system can be disputed in some groups of individuals. For example, for families, it can be helpful to feel that the disease is not limiting the comfort of their life, but on the other hand, they can be more uncertain in situations like damage of the device. This is why constant and repeatable education of patients and their families is always crucial, independently from the device we use in the treatment. It was emphasized in one of our former studies (14).

It is relevant to emphasize that the observed increase in total daily insulin after 2 years of follow-up is mainly the result of the increase in the autocorrection dose. The basal insulin was unchanged after 24 months compared to the baseline. It has been shown that the risk of weight gain increases with the rising percentage of basal insulin in the total daily insulin dose (15). Our findings seem to follow this observation.

The results of the analyzed cohort picture sustained optimal, according to international guidelines, glycemic control when using the AHCL system. It appears that the high use of auto-mode and autocorrection doses contributed notably to these outcomes. Automated insulin delivery is a chance for many children and adolescents with T1D to maintain optimal glycemic control with reduced participation of the patient, his family, and healthcare providers in the treatment. The increase in the use of auto mode and the percentage of autocorrection in total daily insulin doses may indicate an increase in patients’ trust in the system and acceptance of the algorithm.

The study’s strength is that this is the first 2-year-long observation of glycemic outcomes during the use of the AHCL of a pediatric cohort, with initially optimal glycemic control. The number of patients analyzed is also an advantage. The limitations include observing only those parameters typically registered during visits and analysing the data without specific insight into day and night. Another limitation is the lack of data concerning HbA1c levels at the beginning of the study and after 2 years of AHCL treatment.

## Conclusion

Children and adolescents with type 1 diabetes treated with the AHCL system for 2 years maintain adequate weight and optimal glycemic control, most likely due to autocorrection of boluses.

## Data availability statement

The raw data supporting the conclusions of this article will be made available by the authors, without undue reservation.

## Ethics statement

The studies involving humans were approved by Local Bioethics Committee of the Medical University of Silesia in Katowice. The studies were conducted in accordance with the local legislation and institutional requirements. Written informed consent for participation in this study was provided by the participants’ legal guardians/next of kin.

## Author contributions

SS: Conceptualization, Data curation, Investigation, Methodology, Project administration, Writing – original draft, Writing – review & editing. AC: Data curation, Investigation, Supervision, Writing – original draft, Writing – review & editing. MT: Data curation, Methodology, Writing – original draft. AB: Data curation, Writing – original draft. ER: Formal analysis, Methodology, Writing – original draft. AO: Investigation, Methodology, Project administration, Writing – original draft, Writing – review & editing. JP: Data curation, Formal analysis, Methodology, Writing – review & editing. PJ-C: Investigation, Project administration, Writing – review & editing.
